# Egg rejection in blackbirds *Turdus merula*: a by-product of conspecific parasitism or successful resistance against interspecific brood parasites?

**DOI:** 10.1186/s12983-016-0148-y

**Published:** 2016-04-12

**Authors:** Francisco Ruiz-Raya, Manuel Soler, Gianluca Roncalli, Teresa Abaurrea, Juan Diego Ibáñez-Álamo

**Affiliations:** Departamento de Zoología, Facultad de Ciencias, Universidad de Granada, E-18071 Granada, Spain; Departamento de Ecología de Humedales, Estación Biológica de Doñana, CSIC, Sevilla, Spain; Behavioral and Physiological Ecology Group, Centre for Ecological and Evolutionary Studies, University of Groningen, P. O. box 11103, 9700 CC Groningen, The Netherlands

**Keywords:** Interspecific brood parasitism, Conspecific brood parasitism, Egg recognition, Egg rejection, Successful resistance, Common blackbird

## Abstract

**Background:**

Traditional theory assumes that egg recognition and rejection abilities arise as a response against interspecific brood parasitism (IBP). However, rejection also appears in some species that are currently not exploited by interspecific parasites, such as *Turdus* thrushes. Recent evidences suggest that rejection abilities evolved in these species as a response to conspecific brood parasitism (CBP). To test these two alternative hypotheses, we performed an experimental study by parasitizing nests of the common blackbird (*Turdus merula*) with conspecifics or heterospecific eggs under different risk of parasitism (presence of interspecific or conspecific parasites near the nest). Common blackbird is a potential host of the common cuckoo (*Cuculus canorus*) but suffers low levels of CBP too.

**Results:**

We found that blackbirds were able to recognize and eject heterospecific eggs at high rates whereas most of conspecifics eggs were not recognized and, therefore, accepted. Ejection rates of conspecific eggs did not exceed 13 %, even in situations of high risk of CBP (blackbird female placed near the nest), which contradict the main prediction derived from the CBP hypothesis. Conversely, ejection rates of experimental eggs simulating IBP were much higher (80–100 %). Furthermore, female blackbirds were more aggressive towards cuckoos than towards blackbird dummies.

**Conclusions:**

Our results considered together support the IBP hypothesis, indicating that recognition and rejection of parasitic eggs in blackbirds have probably evolved due to previous cuckoo parasitism. The current absence of IBP in blackbirds may be due to the highly efficient rejection abilities in this species. Thus, these abilities have been retained in absence of brood parasitism as a consequence of the low costs involved for blackbirds, resulting in a successful resistance against interspecific brood parasitism.

## Background

Interspecific brood parasitism (IBP hereafter) generally imposes high fitness costs on hosts since the parasitic chick is usually better at competing for food or evicts all host offspring [1, 2]. Under this strong selective pressure, many hosts have evolved defences against brood parasitism operating at every phase of the breeding cycle. Meanwhile, brood parasites have also evolved counter-defences in response to successive stages of host defence, resulting in a coevolutionary arms race between brood parasites and their hosts [1–3].

Rejection of the parasitic egg is the most widespread and effective defence used by hosts against IBP [1]. In response to this, brood parasites have evolved mimetic eggs whose degree of mimicry is related with the strength of host rejection [4, 5]. Therefore, it has usually been assumed that recognition and rejection abilities in hosts arise as a response against IBP [1, 3, 6].

But rejection behaviour also appears in species that are currently not exploited by interspecific brood parasites, which has traditionally been considered as evidence of ancient history of IBP [1, 3, 6]. However, it has also been suggested that conspecific brood parasitism (CBP hereafter; i.e., parasitic females laying eggs in nests of their own species [7]) could also account for egg rejection [8–10]. This argument has been used regarding thrushes in previous studies [8, 11–14]. Samas et al. [14], in an experimental study with two species of *Turdus* thrushes: the common blackbird (*Turdus merula*; blackbird hereafter) and the song thrush (*Turdus philomelos*), concluded that egg discrimination in thrushes has evolved as a response to CBP instead of IBP based on the ejection rate of conspecific eggs found in their study (~20–40 %) and the existence of CBP in their blackbird populations (CBP rates of 3.1 and 0 % in the areas of sympatry and allopatry with the common cuckoo - *Cuculus canorus*; cuckoo hereafter -, respectively). They found that conspecific eggs were ejected more often in the population of higher breeding densities, which is interpreted as a response of blackbirds to the perceived risk of conspecific parasitism. In their work, Samas et al. [14] assume (1) that thrushes are unsuitable hosts that have not been involved in a long-term coevolutionary history with the cuckoo, and (2) that blackbird defences have to decline in the absence of the selection pressures that favoured them (i. e. IBP). Based on these two points, they proposed that IBP is unlikely to be the factor responsible of the evolution of egg rejection in thrushes and suggested that CBP constitutes an evolutionary scenario comparable to IBP that could produce the same antiparasitic adaptations in hosts. This is an important conclusion with great impact in the field of brood parasitism that deserves to be studied in detail. On the other hand, these arguments have been recently discussed by Soler [15], who suggested that conclusions from Samas et al. [14] were based on unclear predictions and, therefore, should be treated with caution. As Soler [15] argued, the evolution of abilities to discriminate and eject conspecific eggs is rare in species that suffer CBP because of two reasons. First, due to the high similarity between host eggs and those laid by the conspecific female, which entails that hosts of conspecific parasites require a more subtle level of recognition than those who are exploited by interspecific parasites, making recognition much more difficult to evolve than in hosts of interspecific brood parasites [16–19]. Second, while IBP imposes dramatic fitness costs to hosts (see above), costs resulting from CBP are much lower, which reduces the strength of selection for defences to evolve [1, 16, 19–21]. In fact, current available information shows that CBP almost never selects for egg rejection: CBP has been documented in 234 avian species [22], but egg rejection has only evolved in a few species. In altricial birds, the evolution and maintenance of rejection defences as consequence of CBP has only been reported in the house sparrow (*Passer domesticus*) [19, 23, 24] and Eurasian tree sparrows (*Passer montanus*) [21], species for which an evolutionary history of relationships with interspecific brood parasites is also likely [19]. Furthermore, there are no reasons to think that the existence of rejection abilities in blackbirds could not have evolved in response to IBP because the maintenance of rejection abilities (successful resistance) in the absence of brood parasitism is a frequent long-term outcome of the relationships between interspecific brood parasites and their hosts [3, 18, 25, 26]. According to calculations in Soler [3], 29.7 % of potential host species that are not currently parasitized reject nearly 100 % of nonmimetic eggs.

In European thrushes, parasitism by the cuckoo was documented in all six species that occur in Europe, but parasitism rates were lower than those in current cuckoo hosts [27], so European thrushes are currently considered not impacted by IBP. Despite this, thrushes species are able to reject foreign eggs from the nest at high rates [12, 28–30] and some species are reluctant to feed cuckoo nestlings experimentally introduced in their nests [12, 31]. Moreover, aggression towards cuckoo dummies has been experimentally demonstrated in thrushes [12, 32], suggesting that IBP was the selective force that selected for egg rejection in this group. Previous studies have classified blackbirds as either suitable [28] or unsuitable host [12] for the cuckoo. Grim et al. [12] concluded that blackbirds were not involved in long-term coevolution with the cuckoo because no cuckoo gens have been found for any *Turdus* species. However, this conclusion is based on an analysis of cuckoo and host eggs from collections of European museums [27] and takes into account a relatively short period of time (only a few centuries) of the interactions between cuckoos and their hosts (tens of thousands of years; [33]). Furthermore, under this scenario, the experimentally demonstrated existence of aggression towards cuckoo dummies and reluctance to feed cuckoo nestlings in thrushes [12] remain unexplained. These defences are especially developed in the blackbird, which attack more frequently a cuckoo dummy than a predator one (49.2 % vs. 33.3 %) and were reluctant to feed even lone cuckoo nestlings [12].

Thus, the origin and maintenance of rejection abilities in thrushes is an interesting evolutionary question that deserves more attention, especially given that previous studies that have addressed this issue did not show conclusive results [14, 34]. Therefore, the main aim of this study is to clarify whether the cause of rejection behaviour in the blackbird is a by-product of conspecific parasitism (CBP hypothesis) or evolved in the past as a defence against interspecific brood parasites (IBP hypothesis). To do so, we carried out an artificial parasitism experiment with blackbirds by manipulating the risk of IBP or CBP simultaneously. Our experimental design expands previous research in two important aspects. First, the risk of IBP or CBP is directly manipulated by presenting a dummy of a cuckoo or a blackbird, respectively. Second, we distinguish between recognition abilities and rejection of the parasitic model eggs. Studies of artificial parasitism focused on discrimination abilities should do such differentiation [35] due to the existence of plastic responses of hosts against the parasitic egg [35–42]. We tested the following predictions on different aspects of antiparasitism defences (see Table 1):

**Table 1:** Summary of predictions derived from IBP and CBP hypotheses. ≤ means a similar or lightly smaller rate

Prediction	IBP Hypothesis (a)	CBP Hypothesis (b)
1 Recognition	CBP eggs < IBP eggs	CBP eggs ≤ IBP eggs
2 General ejection rate	CBP eggs < IBP eggs	CBP eggs ≤ IBP eggs
3 Ejection and risk of parasitism	Higher under IBP risk	Higher under CBP risk
4 Aggression	Blackbird < Cuckoo	Blackbird > Cuckoo

### Recognition of parasitic eggs

**Fig. 1:**
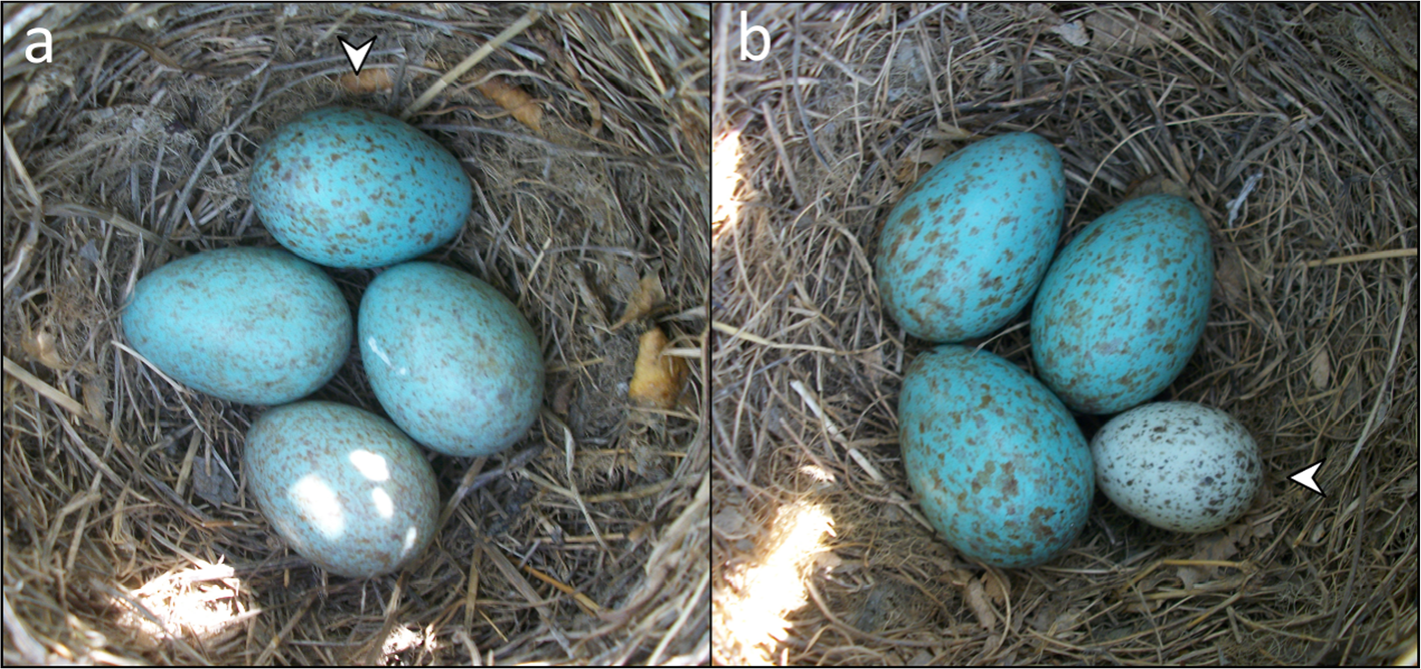
Blackbird nest parasitized with (**a**) conspecific or (**b**) heterospecific egg. Parasitic eggs are indicated with a white arrow

If IBP selected for egg discrimination, then heterospecific eggs (Fig. 1b) should be much better recognized than conspecifics eggs (Fig. 1a) given that a much finer level of discrimination is required to recognize conspecific compared to heterospecific eggs (*Prediction 1a*) [43]. In contrast, (*Prediction 1b*) if CBP selected for egg discrimination, cognitive abilities needed to recognize parasitic eggs evolved in blackbirds as a response to conspecific eggs (highly mimetic eggs both in size and colour). Under this scenario, we predicted that heterospecific (less mimetic) and conspecific eggs should be recognized at a similar level, as occurs in house sparrows (*Passer domesticus*) [24], the only species in which rejection abilities have probably evolved as a consequence of CBP [19]. However, it is well-known that egg recognition is usually conditioned by the degree of egg mimicry [4, 44]. Thus, even if the egg discrimination ability would have evolved under selection from CBP, the non-mimetic heterospecific eggs will probably be more rejected than the highly mimetic conspecific eggs. Therefore, in order to be conservative, our prediction here is that conspecific eggs should be recognized at a similar or slightly smaller rate than heterospecific eggs.

### Ejection of parasitic eggs

(*Prediction 2a*) If IBP selected for egg discrimination, then heterospecific eggs should be ejected at higher rates than conspecifics eggs (see Prediction 1a). Conversely, (*Prediction 2b*) if CBP selected for egg discrimination (i.e., egg recognition have evolved in blackbirds to be able to recognize mimetic eggs) then both conspecific and heterospecific eggs will probably be ejected at similar rates. Moreno-Rueda & Soler [24] found that house sparrow (*Passer domesticus*), a species with CBP, rejects mimetic (i.e., conspecific) and non-mimetic (i.e., heterospecific) eggs at similar rates. However, for similar reasons to those explained in Prediction 1b, our prediction is that conspecific eggs should be ejected at a similar or slightly smaller rate than heterospecific eggs.

### Ejection and risk of parasitism

(*Prediction 3a*) If IBP selected for egg discrimination, ejection rates should be higher in situations of higher risk of IBP. The plastic response of hosts in egg rejection behaviour according to the perceived risk of parasitism (i. e. after they have encountered a cuckoo near their nests) has been documented in many cases [45–48]. However, in this context, it is also predictable that conspecific model eggs are accepted, even in a situation of high IBP risk, if abilities to discriminate conspecific eggs are not fine enough (see Prediction 1a). On the contrary, (*Prediction 3b*) if CBP selected for egg discrimination, ejection rates should be higher in situations of a clear risk of CBP.

### Aggression

(*Prediction 4a*) If IBP selected for egg discrimination, then cuckoo dummies should be more attacked than blackbird or control dummies. Many host species are able to recognize brood parasites near their nests and respond to them aggressively [49–52]. Alternatively, (*Prediction 4b*) if CBP selected for egg discrimination, then blackbird dummies should be more attacked than cuckoo or control dummies.

### Ethical note

The filming of adults or placement of dummies did not cause any negative effect on blackbird egg hatchability relative to natural nests. Research has been conducted according to relevant national (Real Decreto 1201/2005, de 10 de Octubre) and regional (permissions provided by Consejería de Medio Ambiente de la Junta de Andalucía) guidelines.

## Results

We conducted our experiment in 104 blackbird nests. 14 of them were not used in the ejection analyses, because they were predated (11 nests) or deserted (3 nests) before the end of the trial. We assumed that nest desertion is not a response to experimental parasitism in blackbirds [30]. We found two nests where two new eggs were laid per day, so estimated CBP rate in our blackbird population was 2.9 % (*n* = 68).

### Recognition of parasitic eggs

**Fig. 2:**
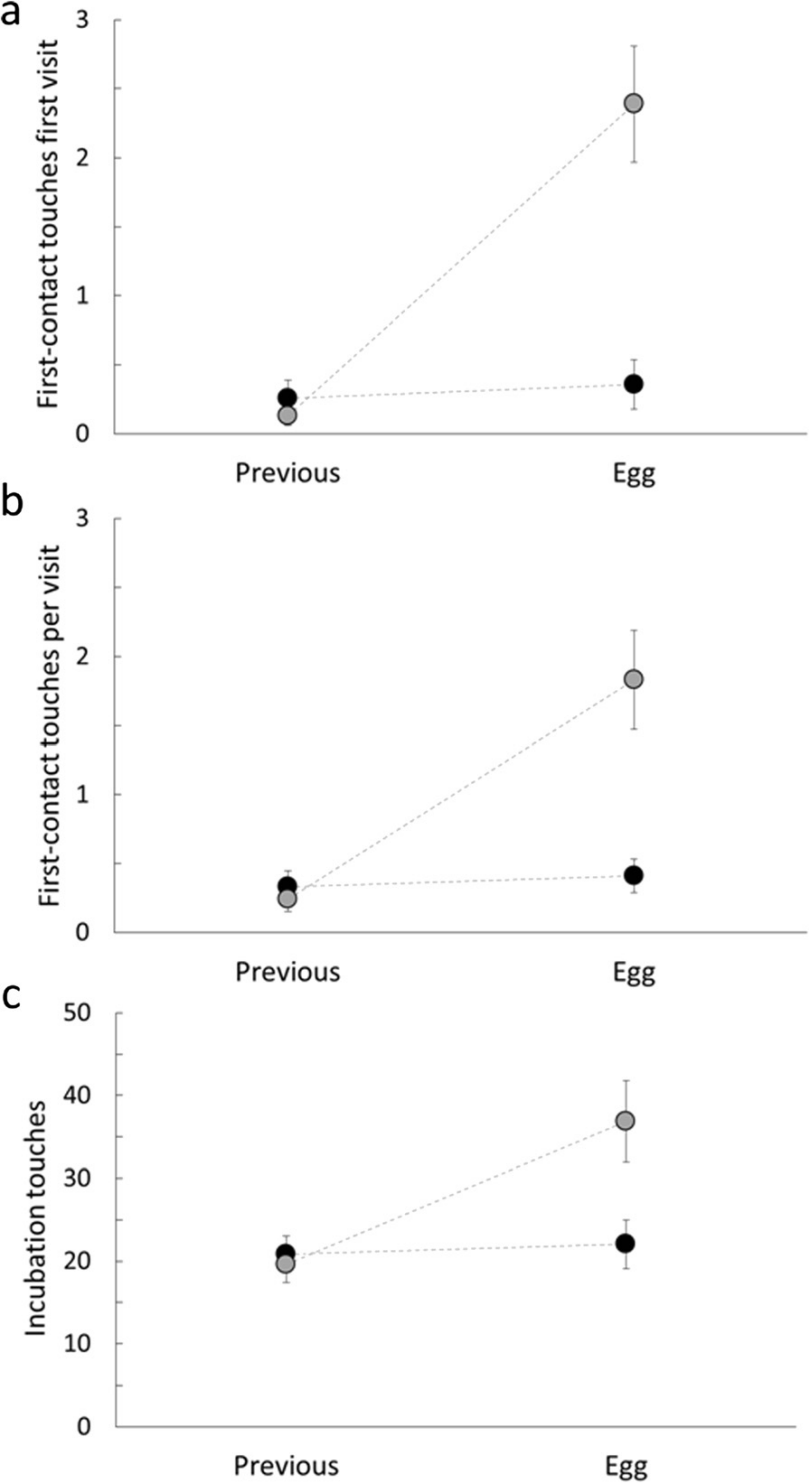
Recognition of conspecific (*black circles*) and heterospecific model eggs (*grey circles*). Differences between “previous session” (before parasitism) and “egg session” (after parasitism) for nests that received either conspecific or heterospecific eggs, regarding: **a** first-contact touches first visit (number of touches in the first visit), **b** first-contact touches per visit (number of touches for all visits corrected by the number of visits) and **c** incubation touches (number of touches during incubation corrected by the incubation time); see Methods section for a more detailed explanation of each variable. We show nests separately (i. e. receptors of conspecific or heterospecific eggs) in the “previous session” in order to clearly illustrate differences between both sessions for the two types of nests. Values are presented as means ± SE

We found differences in recognition between conspecific and heterospecific parasitic eggs in all variables used in our recognition analyses (Fig. 2). Heterospecific model eggs introduced in the nest (“egg session”) were more touched by females than their own eggs (“previous session”) for first-contact touches in the first visit (LRT: *χ*^2^ = 113.6, df = 1, *p* < 0.001; *N* = 104; Table 2; Fig. 2), taking all the visits together (first-contact touches per visit, LRT: *χ*^2^ = 63.59, df = 1, *p* < 0.001) and considering touches during incubation (LRT: *χ*^2^ = 245.1, df = 1, *p* < 0.001). However, females touched conspecific eggs as often as they touched their own eggs for all recognition variables (all cases *p* > 0.5; Table 2; Fig. 2). These results indicate that blackbirds are able to recognize heterospecific but not conspecifics eggs, which supports the IBP hypothesis (Prediction 1a). Other predictors, such as risk of parasitism, did not explain variation in recognition touches by blackbirds for both heterospecific and conspecific parasitic eggs (Table 2).

**Table 2:** Models used from analyses of egg recognition. Results from LRT for the recognition models of the three variables used: first-contact touches first visit, first-contact touches per visit and incubation touches. In all cases, significant predictors are in bold

Recognition	Conspecific brood parasitism			Interspecific brood parasitism		
First-contact touches first visit	df	*χ* ^2^	p	df	*χ* ^2^	p
Dummy	2	0.16	0.92	2	4.10	0.13
Session	1	1.00	0.32	**1**	**113.6**	**<0.001**
D*S	2	4.11	0.13	2	5.00	0.08
clutch size	2	0.46	0.50	2	2.38	0.12
First-contact touches per visit						
Dummy	2	1.44	0.49	2	0.38	0.82
Session	1	0.58	0.45	**1**	**63.59**	**<0.001**
D*S	2	0.92	0.63	2	1.54	0.46
Clutch size	2	0.06	0.80	2	1.29	0.26
Incubation touches						
Dummy	2	0.21	0.90	2	1.40	0.50
Session	1	1.97	0.16	**1**	**245.1**	**<0.001**
D*S	2	4.16	0.12	2	2.86	0.24
Clutch size	2	1.65	0.20	2	0.17	0.68

Considering only the “egg session”, heterospecific eggs were significantly more touched than conspecific parasitic eggs for first-contact touches first visit (t = 3.87, df = 1, *p* < 0.001, *N* = 104), the number of first-contact touches per visit (t = 4.30, df = 1, *p* < 0.001, *N* = 104) and incubation touches (t = 2.09, df = 1, *p* = 0.04, *N* = 104).

### Egg ejection

**Fig. 3:**
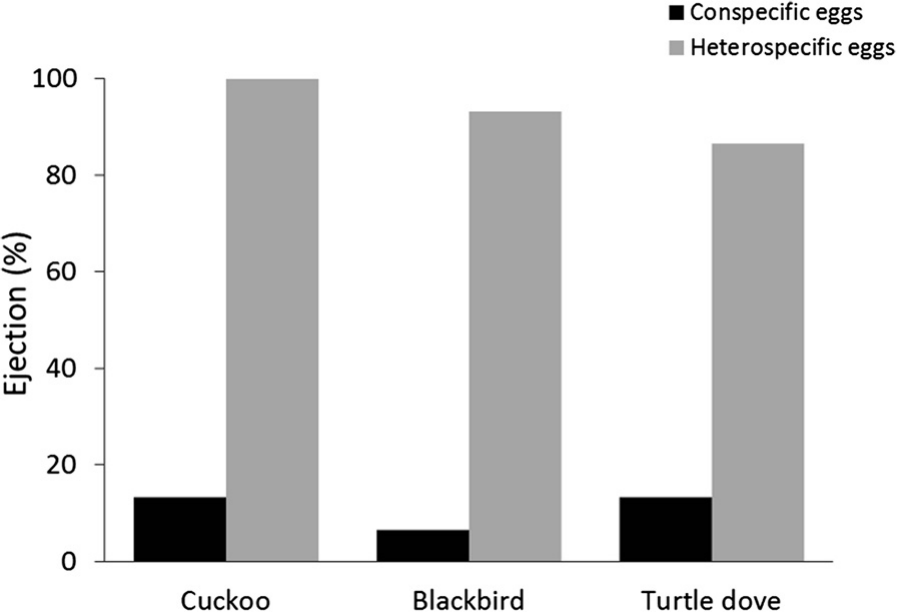
Ejection. Percentage of conspecific (*black*) and heterospecific eggs (*grey*) ejected under different risk of parasitism (presence of a cuckoo, blackbird or a turtle dove dummy)

Model egg (conspecific or heterospecific) was the only predictor that explained the variation in the response of blackbirds to experimental parasitism (GLM: *χ*^2^ = 71.15, df = 1, *p* < 0.001; Table 3; Fig. 3). Thus, heterospecific eggs were significantly more ejected than conspecific parasitic eggs (Tukey HSD: *p* < 0.001), which fits with the IBP hypothesis according to Prediction 2a. Ejection rate of conspecific parasitic eggs was lower than that of heterospecific eggs irrespectively of the risk of parasitism (GLM: *χ*^2^ = 2.63, df = 2, *p* = 0.27, Table 3), which also supports the IBP hypothesis (see Prediction 3a). Clutch size had no effect on blackbird rejection responses to experimental parasitism (see Table 3). No recognition or ejection costs were found in our study.

**Table 3:** Generalized linear model used to test blackbird rejection behaviour to our experimental manipulation. In all cases, significant predictors are in bold

Egg ejection			
	df	*χ* ^2^	p
Egg	**1**	**71.15**	**<0.001**
Dummy	2	1.11	0.57
E*D	2	2.63	0.27
Clutch size	2	2.10	0.15

### Response towards dummies

**Fig. 4:**
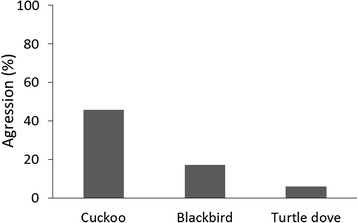
Aggression of blackbirds towards the three different dummies (percentage of cases)

Blackbird responses (in terms of aggression) towards dummies were significantly different depending on the species of dummy placed near the nest (GLM: *χ*^2^ = 91.03, df = 2, *p* = 0.001, *N* = 104). Females were more aggressive towards cuckoo (45.7 % of cases) than blackbird (17.1 % of cases) (Tukey HSD: *p* = 0.049; Fig. 4) or turtle dove dummies (5.9 % of cases) (Tukey HSD: *p* = 0.01). No differences were found between blackbird and turtle dove dummies regarding aggression by blackbirds (Tukey HSD: *p* = 0.49). These results support the IBP hypothesis according to prediction 4a.

The type of dummy placed near the nest also had a significant effect in the “fear” response (see Methods for a detailed explanation) of blackbirds (GLM: *χ*^2^ = 61.68, df = 2, *p* < 0.001, *N* = 104). Thus, females were more often scared in the presence of a cuckoo dummy (31.4 % of cases) than in the presence of a blackbird (2.9 % of cases) (Tukey HSD: *p* = 0.03) or a turtle dove dummy (2.9 % of cases) (Tukey HSD: *p* = 0.03). However, there were no differences for this behaviour between blackbird and turtle dove dummies (Tukey HSD: *p* = 0.1).

Latency of females to arrival was not affected by the type of dummy placed near the nest, either for conspecific (LRT: *χ*^2^ = 1.93, df = 2, *p* = 0.38, *N* = 101) or for heterospecific eggs (LRT: *χ*^2^ = 0.43, df = 2, *p* = 0.81). Furthermore, females did not modify their time at the nest after encountering any dummy near the nest for both conspecific (F = 0.97, ddf = 53.99, *p* = 0.39, *N* = 88) and heterospecific eggs (F = 0.78, ddf = 28.00, *p* = 0.47). In contrast, nest checking behaviour was affected by the type of egg introduced, and partially by the type of dummy presented. We found a significant increase in nest checking in nests parasitized with heterospecific eggs (LRT: *χ*^2^ = 32.32, df = 1, *p* < 0.001, *N* = 88) regardless of the type of dummy placed near the nest (LRT: *χ*^2^ = 3.18, df = 2, *p* = 0.2). Regarding those nests parasitized with conspecific eggs, we found a significant increase in nest checking only in those females who had encountered a cuckoo dummy (LRT: *χ*^2^ = 5.87, df = 2, *p* = 0.05).

## Discussion

The origin and evolution of rejection abilities in species that are not frequently exploited by interspecific brood parasites has been previously addressed in several studies (e. g., [8, 11–14]). In this work, we experimentally tested for the conspecific or interspecific origin of the rejection behaviour in the blackbird by artificially parasitizing natural nests with conspecific or heterospecific eggs under different risk of parasitism. However, since recognition is not always followed by rejection of the parasitic egg [35, 38, 40, 42, 53], it is necessary to conduct experimental studies that provide information on both rejection and discrimination abilities. Regarding recognition, heterospecific eggs were easily recognized by females from the first contact with the parasitic egg. Female blackbirds touched the heterospecific model eggs repeatedly both on arrival at the nest and during incubation (Fig. 2) indicating egg recognition. However, when nests were parasitized with conspecific eggs, females did not recognize them during the two first hours after the experimental parasitism, which supports the IBP hypothesis according the Prediction 1a. Clearly, the high similarity between host eggs and conspecific model eggs makes difficult the recognition of the latter. This supports the previously suggested idea that discrimination abilities are much more difficult to evolve in hosts suffering only CBP than in those parasitized by heterospecifics (see above).

The initial absence of recognition of CBP eggs was confirmed in the ejection rate, since acceptance was the most common response against conspecific parasitic eggs (Fig. 3). Consequently, conspecific eggs were ejected at much lower rates (less than 13 %) compared with ejection rates of heterospecific eggs (nearly 100 %), which also supports the IBP hypothesis (Prediction 2a). Although the CBP hypothesis may also predict a higher ejection rate of heterospecific than conspecific eggs (see Prediction 2b above), the very low ejection rates of conspecific eggs found in our study do not support the CBP hypothesis. This is not striking because experimental studies have shown that many rejecters of IBP eggs often show low or no ability to reject CBP eggs ([28, 54, 55], but see [26, 56]). In our study, the few cases of ejection of a conspecific egg could be explained by the fine ability of blackbirds to recognize IBP eggs, which would enable them to recognize some CBP eggs, probably those less similar to their own eggs, in terms of colour or shape, as occurs in some species (e. g., [57]). Regarding ejection rate and the perceived risk of CBP or IBP, we found that conspecific eggs were systematically ejected at low rates regardless of the presence of a blackbird dummy (Fig. 3), contradicting Prediction 3b derived from the CBP hypothesis. Conspecific model eggs were also ejected at low rates in the presence of a cuckoo dummy, which could be expected according the IBP hypothesis (Prediction 3a) since, in this context, recognition of conspecific eggs is more difficult to evolve (see Prediction 1a). In fact, blackbirds were usually not able to discriminate against conspecific eggs (see above). Taken together, these results fit again the IBP hypothesis. The low ejection rates of conspecific eggs found in our study contrast with those found by Samas et al. [14], further considering that our blackbird population presents a three times higher density (2.9 pairs/ha, [58]) than those used by them in their predictions of high density populations in New Zealand [14] (1 pair/ha max, [59]), which should increase the rejection of conspecific eggs by hosts in our study area [14, 60]. This results calls into question the use of indirect information on breeding density for manipulating the risk of IBP or CBP, which should be done by presenting directly dummies of cuckoo or blackbirds, respectively. The higher ejection rates of conspecific eggs found by Samas et al. [14] could be explained by a population bottleneck during the blackbird’s introduction in New Zealand or due to a higher difference in inter-individual egg variability in their population, which would facilitate recognition of conspecific eggs. Clearly more studies are needed to clarify these differences between populations. Finally, the absence of differences in ejection of heterospecific model eggs despite the presence of a cuckoo near the nest could be due to the high ejection rates in all cases (close to 100 %). In fact, strong ejection of heterospecific eggs has been previously found in other experimental studies in blackbirds [12, 14, 29, 30, 35, 61].

We found a particularly aggressive response of blackbirds towards cuckoo dummies (Fig. 4), indicating that they were perceived by females as an important risk of parasitism [49–52, 62] and supporting the IBP hypothesis according with the Prediction 4a. Thus, in our study, aggressions were specifically directed towards cuckoo dummies, which suggest that in this case the blackbird behaviour is a response to the threat presented by the parasite and not a result of generalized nest defence, as has been suggested in previous studies [63]. Although we can predict the existence of plasticity in host behaviour towards cuckoo dummies regarding allopatry or sympatry with the parasite [32], the aggression rates found in our study are similar to those found for other potential cuckoo hosts, including some of the most common hosts [64]. Furthermore, the aggressive response towards cuckoo dummies found in our study area is not surprising because it has also been reported in other blackbird populations [12, 63]. This is so, even after considering the scary effect of the cuckoo-hawk mimicry, which usually reduces the aggression to cuckoos by hosts [49, 65]. Despite this cuckoo-hawk mimicry, cuckoo dummies were usually perceived by females as a brood parasite instead of predator (i. e. sparrowhawk) as the time spent by females at the nest did not decrease and the latency to arrival did not increase specifically in the presence of a cuckoo dummy, as occurs in other unusual hosts after encountering a predator near the nest [62]. Furthermore, we found a specific increase in the time the females spent checking their nests after encountering a cuckoo dummy in those nests parasitized with conspecific eggs. In the case of parasitism with heterospecific eggs, females increased their time checking the nest regardless of the type of dummy. These results seem to indicate that recognition of a parasitic egg alerts the females and they spend more time inspecting the clutch; but it also mean that, even if the parasitic egg is not recognized, the presence of a cuckoo near the nest is perceived by blackbirds as a specific threat of parasitism. Interestingly, fear behaviour was also a frequent response of females towards cuckoo dummies, but not towards blackbird or turtle dove dummies, which may be explained in these cases by the cuckoo-hawk mimicry. However, in these cases, the shock was limited to the first visual contact with the cuckoo dummy, as blackbirds often remained in the nest area and did not delay the arrival to the nest compared to the turtle dove or blackbird dummies. Previous studies have shown that some species delay their return to the nests after perceiving a risk of predation [66], which does not occur in the blackbird after encountering a cuckoo dummy. We also reported some cases of aggressive behaviour towards blackbird dummies, although significantly less than towards cuckoo dummies (Fig. 4). In these cases, intraspecific territoriality may explain the response towards blackbird dummies if females perceived them as potential competitors for food or nest sites [67], especially considering the high breeding density in our population (see above).

Although some previous studies considered the blackbird as an unsuitable cuckoo host [12, 14, 63], our results suggest that the existence of rejection abilities in blackbirds probably evolved in response to IBP as a consequence of historical interactions with the cuckoo. Cuckoos are not currently present in our study area, but the unmistakable song of cuckoo males was frequently heard only 30–40 years ago according to locals living in the area. Given that real unsuitable host species such as those breeding in inaccessible nest sites or those feeding nestlings with seeds are pure acceptor species [28, 68], rejection of foreign eggs by thrushes is an indication of past parasitism [1, 3, 6]. The patterns found by Grim et al. [12] (see above) can be understood under the IBP hypothesis taking into account that reciprocal adaptations between brood parasites and their hosts occurring at all stages of the breeding cycle and that different lines of defence can evolve in all those stages [2, 3]. Thus, current absence of parasitism in blackbirds may be due to the highly efficient rejection ability in this species, which would provoke the cuckoo to switch to other host species with less developed defences [3]. As a matter of fact, most currently non-parasitized potential hosts of the cuckoo show a rejection rate of nearly 100 % [27–29], which has been retained over very long time periods including speciation events [18, 25, 69], even in the absence of CBP [69–73]. Soler [3], showed that about 30 % of potential host species of brood parasites present an ejection rate of nearly 100 %. In the case of blackbirds, non-mimetic eggs are ejected at high rates and, in many cases, nearly 100 % ([12, 28–30], this study). Moreover, many of the rarely used potential hosts of the cuckoo show high rejection rates of non-mimetic eggs, in many cases higher than current frequently used hosts [28, 68, 74–76].

According to traditional theory, costs associated with maintaining the non-functional traits will determine the persistence of such traits for long periods of time [77]. Thus, the maintenance of egg rejection in species that are currently not affected by IBP has been considered in many cases as an evolutionary enigma because a rapid decline of rejection abilities would be predicted [78]. This decline of the rejection behaviour would give rise to coevolutionary cycles that would allow parasites to return later to the previous population [79] or host species [28, 80, 81]. Although several experimental studies have shown evidences of rejection and recognition costs in some species [14, 44, 81–83], the absence of such costs is the rule instead of the exception [3]. This is also true for the blackbird: one study has reported low costs [14], but all others have found absence of costs ([30, 35, 84, 85], this study). Furthermore, only recognition errors in non-parasitized nests would select for the loss of egg recognition abilities [36] and this type of error has only been reported once in blackbirds [14], but not in others studies in this species ([30, 35], this study) or in any other host species [17, 86–88] . Therefore, if there are no costs for maintenance of the rejection abilities, brood parasite-host coevolution might result in successful resistance, preventing future exploitation of host species by parasites [1, 3]. Indeed, successful resistance is a very frequent outcome of brood parasite-host interaction and high rejection rates are maintained in some potential host species that are currently not exploited by interspecific parasites [reported in 54 host species (29.7 %) [3]].

Recognition and rejection of parasitic egg is a widespread defence used by host against IBP [1] but is absent in most species suffering CBP. The existence of CBP in the blackbird populations used by Samas et al. [14] is considered as one of the lines of evidence supporting their conclusion that egg rejection evolved in response to CBP instead of IBP. However, their reported rates of CBP in blackbirds (3.1 and 0 % in sympatric or allopatric areas with the cuckoo, respectively) are extremely low according to theoretical predictions, as occurs in our study area (2.9 %) and other blackbird populations (3.9 and 5.0 %, [58]). Could percentages of CBP of this magnitude support the hypothesis that egg recognition evolved to counter CBP? To answer this question we used the Davies et al.’s [36] signal-detection model. This methodology allowed Underwood et al. [73] to estimate the level of CBP necessary to select for conspecific egg rejection in the black-billed magpie (*Pica hudsonia*); specifically, the values of CBP predicted by the model were 22–49 % (based on two different assumptions). In the case of blackbirds, even assuming fairly high costs for CBP (i. e. the loss of a chick in a parasitized nest) and considering the rejection costs found by Samas et al. [14], the signal detection model predicts that values of CBP occurrence needed for the evolution of responses against conspecific eggs in the blackbird would range from 55 to 65 %. Thus, it can be concluded that extremely low CBP rates reported in blackbirds (see above) do not support that CBP is an important pressure favouring the evolution of egg discrimination. Furthermore, it deserves to be emphasized that rejection based on discrimination is absent in most species suffering CBP, including those with a high frequency of CBP. For instance, in cliff swallows (*Hirundo pyrrhonota*) CBP is detected in about 24 % of nests but egg rejection occurs only when the experimental egg is added before the host female has laid its first egg, but never thereafter [89]; which also occurs in other species that readily reject conspecific model eggs [90]. This indicates that absence of rejection is based on a lack of egg recognition instead of a physical impairment to reject conspecific eggs [20].

## Conclusions

Our results fitted all predictions based on the IBP hypothesis but none of those based on the CBP hypothesis. Female blackbirds recognized easily heterospecific but not conspecific eggs. As occurs in most of non-parasitized potential hosts of the cuckoo, blackbirds showed a high ejection rate (independent of perceived risk of parasitism) and, furthermore, high aggression towards cuckoo dummies, suggesting a historical interaction between these two species. In addition, CBP occurrence in natural blackbird populations is well below the expected theoretical levels that will allow for the evolution of such defenses due to CBP alone. Finally, current absence of cuckoo parasitism in the common blackbird may be the consequence of the very low recognition and ejection costs found in this species, which will result in the maintenance of antiparasitic defences, leading to successful resistance. All of these pieces of evidence together strongly suggest that the evolutionary origin of egg recognition and rejection abilities in this species has probably been cuckoo parasitism.

## Methods

### Study site and species

We carried out experiments in the Valley of Lecrín (Southern Spain, 36° 56′ N, 3° 33′ W; 580 m a.s.l.) from March to May 2014. The study area is dominated by orange groves, in which blackbirds usually nest. For a detailed description of the population, see [91]. The common cuckoo is not currently present in the study site but there are evidences of their presence in the area until thirty years ago (personal information).

The blackbird, one of the most common thrushes in Europe, is a potential host species for cuckoos, but it is currently rarely parasitized [12]. This species has frequently been used as a model species in egg-recognition experiments (e.g., [11, 12, 14, 29, 30, 35, 61]), which have provided us detailed information about their response to experimental foreign eggs.

### Experimental procedure and data collection

We actively searched for blackbird nests in the study area throughout the breeding season of 2014. Once a nest was located, we checked it using a mirror to determine its content. The nest was visited every two days to obtain data on laying date and clutch size.

To determine blackbird responses to parasitism, nests were experimentally parasitized and parents were exposed to different parasitism risk situations. We created six different treatments by combining two factors: type of parasitic egg (conspecific or heterospecific) and risk of parasitism (risk of CBP, risk of IBP and control). Each nest was randomly assigned to one of these six treatments.

CBP was simulated by introducing a real conspecific egg from previously deserted nests of the same population (Fig. 1a). To simulate IBP, we used real house sparrow (*Passer domesticus*) eggs from deserted nests of a captive population maintained at the University of Granada (Fig. 1b). The use of real eggs exclude the potential problems of other types of model eggs used in artificial parasitism experiments (i.e., clay, plasticine…) like an increased costs of rejection and/or nest desertion [84]. Furthermore, House sparrow eggs are similar in size to cuckoo eggs from the south of Spain [84]. We used non manipulated real eggs of house sparrows to ensure that all parasitized nests (CBP and IBP) were in the same conditions. Eggs were introduced into the nests during the laying (minimum of two eggs laid) or incubation (at most the ninth day from the onset of lay) stages and each nest was tested only once. Previous studies have shown that blackbirds reject experimental eggs at similar rates in both laying and incubation stages (e.g., [11, 12, 28, 92]). Before experimentally parasitizing a blackbird nest, we numbered all eggs near the blunt pole using a non-toxic marker; previous studies have shown that marks on the blunt pole does not affect host responses to eggs [13, 14, 93].

We placed a video camera (Panasonic HDC-SD40) near the nest (2 – 2.5 m) and filmed normal blackbird behaviour for 1.5 h before egg introduction (“previous session”). After the experimental parasitism, we continued filming for the following two hours (“egg session”) in order to record the blackbirds’ responses to the parasitic egg and their nest attendance. We followed a standardized procedure previously used in other studies with this species [30, 35, 94]. The video camera was placed as high as possible in order to film all the eggs; unfortunately, this was not possible in all cases so we used differences in touches between “previous” and “egg” session to determine the recognition of the parasitic model eggs. The placement of a camera near the nest did not affect blackbird behaviour in relation to egg-recognition experiments [30, 35].

Immediately after introducing the parasitic egg, a painted wooden dummy was placed near the blackbird nest (2–3 m) in order to simulate a risk of parasitism. In all cases we ensured that dummies were easily seen from the nest and the surrounding area. We used female cuckoo (simulating risk of IBP), blackbird (simulating risk of CBP) or turtle dove dummies (*Streptopelia turtur*, control). The turtle dove is a frequent species in our study area that shows a neutral interaction with the blackbird (pers. obs.). We used three specimens of each dummy type. Blackbird responses did not differ between specimens, so we pooled the data (results not shown). After placing the dummy close to the blackbird nest, one of us hid in the area with a camouflage tarpaulin and observed the response of the focal female blackbird towards dummies for 5 min after she appeared in the vicinity of the nest and became aware of the dummy. We noted the latency to the first arrival and the minimum distance from the dummy. The reaction of blackbirds was noted following the scale (from 1 to 4) proposed by Moksnes et al. [64]: (1) “No reaction”, when females remains near the nest ignoring the dummy and even returned to the nest in some cases and began to incubate the eggs, (2) “distress calls”, when blackbird stay in the area and uttered distress or alarm calls, (3) “mobbing”, when females performed flights around the dummy or dives close to it but without touching it, and (4) “attack”, when blackbird attacked the dummy with a strong contact. Following the methodology used by Røskaft et al. [32], we pooled “no reaction” and “distress calls” behaviours as “no aggression”, and “mobbing” and “attack” behaviour as “aggression”. We scored one additional response as “fear” when the blackbird suddenly left the area of the nest obviously frightened after seeing the dummy, in some cases with a strong alarm call. We considered “fear” as “no aggression” for the aggression analyses. We presented only one type of dummy (cuckoo, blackbird or turtle dove) near each nest. To standardize this data, all observations were made by the same author (FRR).

After the two recording hours, we checked for the introduced egg. If the model egg remained in the nest, we checked it again after 24 h and continued visiting the nest for the following five days to determine the ejection time. This five-day period has been used in other egg-rejection experiments conducted in thrushes (e.g., [11, 30, 35, 61, 92, 95]). When the model egg disappeared we assigned the ejection time considering that the ejection occurred between the last two visits adding 12 h to the time (in hours) of the last visit in which the introduced model egg was still present. We considered the egg was “accepted” if it remained intact at the nest for five days after its introduction. All eggs (blackbird and introduced) were inspected during each visit to look for possible cracks or broken eggs (ejection costs) or mistakenly ejected eggs (recognition costs). We estimated the CBP rate in our population from those nests found during the nest building or laying stage (one egg) by checking these nests every day during the laying stage in order to find cases in which two eggs were laid per day [14].

### Variables and statistical procedures

We used the recordings to extract information related to nest attendance and egg recognition. We analysed three different variables to assess the nest attendance of females after encountering the dummies: (1) the time taken by the females to return to the nest (latency), (2) time that females spent at the nest per hour (time at the nest) and (3) time spent by females inspecting the nest, corrected by the time spent at the nest (nest checking). Regarding egg recognition, we used three variables following the procedure of Ruiz-Raya et al. [35]: (1) “first-contact touches first visit” (number of times the female touched the eggs with its bill from her arrival to the nest until she sat on the nest for the first visit), (2) “first-contact touches per visit” (similar to the previous variable but for the complete filmed period corrected by the number of visits) and (3) “incubation touches” (number of times the female touched the eggs with its bill during interruptions of incubation corrected by the incubation time). For analysis, we utilized mainly Generalized Linear Mixed Models (GLMM) by using *lme4* (R package v.1.1-10 [96]). We included female identity as random factor and the following predictors: dummy (species of dummy placed near the nest), session (before and after the experimental parasitism), D*S (interaction between dummy and session) and clutch size (number of eggs in the nest during the trial). Conspecific and heterospecific model eggs were analysed separately and Laplace approximation of likelihood was used for the parameter estimation. This approach does not allow F-test for fixed effects, so we report the *χ*^2^ statistics from the likelihood ratio test (LRT) between models. We performed an additional analysis to assess differences in recognition between conspecific and heterospecific model eggs considering only the “egg session”. To do this, we performed a negative binomial generalized linear model (GLM) by using *MASS* (R package v.7.3–45 [97]) in order to deal with overdispersion. Time at the nest was analysed by using Linear Mixed Model (LMM). In this case, we adjusted our model by REML using the *lme4* R package and checked the model assumptions.

To assess the response of females to experimental parasitism (ejection) we used Generalized Linear Models (GLM’s: binomial error and logit link function). We included the following predictors in the model: egg, dummy, E*D (interaction between egg and dummy) and clutch size. We also used GLM’s (binomial error and logit link function) to analyse the response of female towards dummies regarding aggression (aggression or no aggression, see above) and fear (yes or no). Differences between levels were compared by using *multcomp* (R package v.1.4-1 [98]. All analyses were performed using R version 3.1.1 [99].
